# Vertebral artery dominance contributes to basilar artery curvature and peri-vertebrobasilar junctional infarcts

**DOI:** 10.1136/jnnp.2008.169805

**Published:** 2009-05-03

**Authors:** J M Hong, C-S Chung, O Y Bang, S W Yong, I S Joo, K Huh

**Affiliations:** 1 Department of Neurology, Ajou University Medical Centre, Suwon, South Korea; 2 Department of Neurology, Samsung Medical Centre, Sungkyunkwan University School of Medicine, Seoul, South Korea

## Abstract

**Objectives:**

The diameters of the vertebral arteries (VAs) are very often unequal. Therefore, this study investigated if unequal VA flow contributes to the development of basilar artery (BA) curvature and if it is a link to the laterality of pontine or cerebellar infarcts occurring around the vertebrobasilar junction.

**Methods:**

Radiological factors were analysed (infarct laterality, VA dominance, BA curvature and their directional relationships) in 91 patients with acute unilateral pontine or posterior inferior cerebellar artery (PICA) territory infarcts. The “dominant” VA side was defined as either that the VA was larger in diameter or the VA was connected with the BA in more of a straight line, if both VAs looked similar in diameter on CT angiography. Multiple regression analysis was performed to predict moderate to severe BA curvature.

**Results:**

The dominant VA was more frequent on the left side (p<0.01). Most patients had an opposite directional relationship between the dominant VA and BA curvature (p<0.01). Pontine infarcts were opposite to the side of BA curvature (p<0.01) and PICA infarcts were on the same side as the non-dominant VA side (p<0.01). The difference in VA diameters was the single independent predictor for moderate to severe BA curvature (OR per 1 mm, 2.70; 95% CI 1.22 to 5.98).

**Conclusions:**

Unequal VA flow is an important haemodynamic contributor of BA curvature and development of peri-vertebrobasilar junctional infarcts.

Atherosclerosis is a chronic, inflammatory, fibroproliferative systemic disease primarily of large and medium sized conduit arteries.^1^ Atherosclerotic plaques tend to develop in focal regions with complicated flow patterns, or with low or oscillatory wall shear stress (WSS) as in regions such as bifurcations, bends and junctions.^2^ ^3^ ^4^ Mechanical forces acting on the arterial wall, including the WSS, are thought to be local factors influencing the development of atherosclerosis and regulating vessel calibre and morphology (ie, vascular remodelling).^5^

The basilar artery (BA), which supplies the brainstem and posterior part of the human brain, arises from the junction of the two vertebral arteries (VAs). Unlike most systemic arteries, which have a tree-like branching pattern, the BA is the only large artery in which two arterial flows merge. Few studies have examined the flow dynamics at the vertebrobasilar junction.^3^ ^6^ In addition, the diameters of the VAs are of equal size in only 6–26% of patients in angiographic or postmortem studies, and the left VA is often larger than the right VA.^7^ Therefore, we postulated that the unequally mechanical forces resulting from asymmetric VA flow might influence the morphological deformation in the vertebrobasilar arterial system (lateral displacement or elongation of the BA, hypoplasia of the VA), and by extension, such deformations might asymmetrically induce the development of infarcts in the areas before or after the vertebrobasilar junction.

To test this hypothesis, we investigated the demographics, specific locations of unilateral infarcts around vertebrobasilar junction, radiological findings and haemodynamic findings. We also analysed the potential predictors of the moderate to severe BA curvature.

## Patients and methods

### Patients

From January 2005 to June 2007, we retrospectively identified all consecutive acute ischaemic stroke patients admitted to two tertiary referral medical centres. We included patients if they had a final diagnosis of acute ischaemic stroke with diffusion weighted imaging (DWI) confirmation of lesions involving the posterior inferior cerebellar artery (PICA) territory unilaterally or pons unilaterally. A detailed history of vascular risk factors was obtained from each patient. To identify the potential mechanism of cerebral infarcts, a set of diagnostic tests was performed that included CT angiography, routine blood tests and a cardiology workup (ECG and echocardiogram). MRI was performed in all patients with a 1.5 T scanner (GE Medical, USA) and scanning with conventional T2 weighted MRI and DWI was conducted in the axial plane using 7 mm thick sections.

Based on the results of the vascular and cardiology studies, we divided the patients’ stroke mechanisms into six groups: cardioembolism, large artery atherosclerosis, small artery disease, other determined aetiologies (eg, vasculitis or dissection), undetermined aetiology and coexisting aetiologies (>1 cause). We excluded patients: (1) if their mechanisms fell into the cardioembolism, other determined cause or coexisting aetiology group, other determined cause or coexisting aetiology; (2) if the VA or BA was not seen on angiographic study; or (3) if the pontine lesion coexisted with the PICA territorial lesion.

To compare the vascular anatomical status (the morphology of the BA and VA) of patients with posterior circulation ischaemic stroke with those of age matched populations, we evaluated brain CT angiography of those patients aged over 40 years with no history of stroke who visited the outpatient clinic of the Department of Neurology, Ajou University Hospital from January 2007 to June 2007. The study was approved by the Institutional Review Board at Ajou University Hospital.

### Imaging analysis

Topographical determination of acute unilateral cerebellar infarction in the PICA territory was performed using visual correlation between Amarenco’s templates and the locations of high signal intensities from DWI that were more than 2 cm in diameter.^8^ Two of the authors (JMH and CSC) came to a topographical consensus. We defined acute unilateral pontine infarction as DWI lesions involving the pons unilaterally. To evaluate the frequency of affected sites in the cerebellum and pons, we made contour maps using MRIcro software (C Rorden, www.mricro.com).

The diameter of each vessel was calculated as the average of the measurements made at three consecutive points, 3 mm apart, starting from the vertebrobasilar junction (both VAs and the BA). The “dominant” VA was defined as (1) having the larger diameter within a strict criterion for diameter (ie, a side to side diameter difference ⩾0.3 mm)^7^ or as (2) the VA connected to the BA in a more straight fashion if both VAs were visually similar to a criterion of angle on CT angiography.

The direction of BA curvature was designated as “right (R)” or “left (L) side” according to a course of BA navigation at the vertebrobasilar junction. The degree of BA curvature was evaluated using a previously suggested CT based method,^9^ based on the lateral-most position of the BA throughout its course (0, midline; 1 (R or L), medial to lateral margin of the clivus or dorsum sellae; 2 (R or L), lateral to the lateral margin of the clivus or dorsum sellae; and 3, in the cerebellopontine angle cistern). Moderate to severe BA curvature was defined as ⩾2 of the above criteria. The height of the bifurcation of the BA was scored as:1, within the suprasellar cistern; 2, at the level of the third ventricle floor; and 3, indenting and elevating the floor of the third ventricle.

### Transcranial Doppler and wall shear stress analysis

Transcranial Doppler was performed with a 2 MHz pulsed Doppler instrument (EME TC-8080, USA) by an experienced technician within 7 days of admission. Patients were asked to relax and breathe normally while in a comfortable supine position in a quiet room. BA flow was identified by the signal directed away from the probe through the suboccipital window and was evaluated at a depth between 80 and 100 mm through a suboccipital window. After the probe was shifted a few centimetres inferolaterally, it detected VA flow signals directed away from the probe at depths of 40–75 mm.

Based on the assumption of laminar blood flow in the setting of a Poiseuillian parabolic model, we calculated the mean wall shear rate (WSR): WSR  =  4× mean blood flow velocity/internal diameter.^10^ WSR was also calculated for mean shear rate. Whole blood viscosity (*η_w_*) was estimated from general plasma viscosity at 37°C (*η_0_* = 1.5 *cP*), and haematocrit (*Ht*) of the patients was determined using Einstein’s equation [*η_w_ = η_0_* (1+2.5 *Ht*)]. Mean wall shear stresses were calculated using the formula: WSS  =  *η_w_* × WSR.^11^ ^12^

### Statistical analysis

Differences between groups (patients vs controls, right vs left, dominant vs non-dominant VA side or each tertile according to the diameter difference of the VAs) were analysed using the Student’s t test, χ^2^ test, paired t test or analysis of variance, as appropriate for continuous and categorical variables. We performed a multiple regression analysis to determine which variables were independent predictors of moderate to severe BA curvature. All potential predictors were entered into a univariate logistic regression model, including demographic variables (ie, age, sex and risk factors for stroke) and radiological/haemodynamic variables (ie, R or L VAs diameter, BA diameter, diameter difference of VAs) and WSS (ie, dominant VA, non-dominant VA and BA). Potential factors that were not significant (p>0.2) in the univariate analysis were sequentially deleted from the full multivariable model. Results are given as odds ratio (OR), as estimates of the relative risk with 95% confidence interval (CI). Statistical significance was considered at p<0.05.

## Results

### General demographics and radiological and haemodynamic findings

Of 133 consecutive patients with an acute unilateral pontine or PICA infarction, 91 were eligible for this study. Forty-one patients were excluded for cardioembolism (n = 26), invisible VA or BA (n = 11), VA dissection (n = 3), coexistence of pontine and PICA infarcts (n = 1) and BA dissection (n = 1). Mean age of the patients was 63.6 (11.6) years and 55 (60.4%) were men.

Table 1 shows the comparative results for age, sex and radiological variables between 83 controls and 91 patients. There were no significant differences in age or sex, direction of dominant flow VA (predominantly left-sided) or diameter of respective arteries (right and left VA, and BA) between cases and control; however, there were significant differences in the degree of BA dolichosis and BA height between the two groups. Mean viscosity was 3.06 (0.17) Cp (centipoise, range 2.57–3.45 Cp) in patients. Mean WSS of the VAs was significantly higher on the right side than on the left (10.4 (6.8) vs 8.0 (5.2) dyn/cm^2^; p = 0.009), especially on the non-dominant flow VA side (11.3 (7.4) vs 7.1 (3.4) dyn/cm^2^; p<0.001). On the contralateral side, no difference was observed in mean WSS according to BA depth between 80 and 90 mm (7.6 (4.4) vs 7.6 (4.7) dyn/cm^2^; p = 0.965).

**Table 1:** Clinical and radiological characteristics of controls (n = 83) and patients (n = 91) with unilateral pontine or PICA infarcts

	Controls (n = 83)	Patients (n = 91)	p Value
General demographic data			
Age (year) (mean (SD))	62.5 (11.7)	63.6 (11.6)	0.526
Sex (% men)	49 (59.0)	55 (60.0)	0.850
Risk factors (n (%))			
Hypertension		65 (71.4)	
Diabetes mellitus		35 (38.5)	
Smoking		30 (33.0)	
Previous stroke		18 (19.8)	
Hyperlipidaemia		22 (24.2)	
Direction of vascular deformations			
Direction of dominant flow VA (n)			0.803
R side	27	28	
L side	56	63	
Direction of BA curvature (n)			0.007
R side	45	68	
L side	29	21	
Visually straight BA	9	2	
Degree of BA curvature (n)			<0.001
Grade 0	9	2	
Grade 1	65	41	
Grade 2	9	35	
Grade 3	0	13	
Degree of BA height (n)			<0.001
Grade 1	82	73	
Grade 2	1	17	
Grade 3	0	1	
Diameter of arteries (mm)			
BA (mean (SD))	3.17 (0.70)	3.20 (0.56)	0.759
R VA (mean (SD))	2.35 (0.82)	1.95 (0.68)	0.001
L VA (mean (SD))	2.48 (0.72)	2.48 (0.74)	0.998

BA, basilar artery; L, left; PICA, posterior inferior cerebellar artery; R, right; VA, vertebral artery.

### Directional relationships of the radiological findings

**Figure 1:**
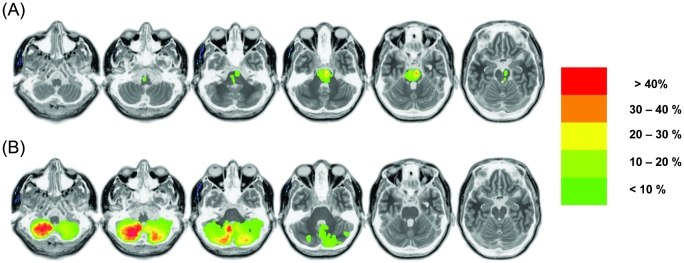
Overlapping contour maps of peri-vertebrobasilar junctional infarcts. Paramedian region of the left mid-pons was the most affected site for post-vertebrobasilar junctional infarcts (ie, pontine infarcts) whereas the right inferior medial region in the cerebellum was the most affected site for the ischaemic infarcts around the pre-vertebrobasilar junction (ie, posterior inferior cerebellar artery infarcts).

The contour maps of the affected sites in the pons and PICA cerebellum are depicted in fig 1. We observed 47 pontine infarcts (R 20 vs L 27) and 44 PICA infarcts (R 26 vs L 18). For pontine infarcts, the paramedian region of the left mid-pons was the most affected site whereas the right inferior medial region in the cerebellum was the most affected site for the PICA infarcts.

The dominant VA side was determined by the diameter criteria in 84 (92.3%) patients, and the rest of the patients by criteria of angle. The dominant VA was more frequent on the left VA side (69.2%; p<0.001). BA curvature was mainly directed to the right side and the most frequent morphological change in the BA was a C-shaped deformation (n = 65), followed by S-shaped (n = 17), J-shaped (n = 7) and no deformation or straight (n = 2). Forty-eight patients had moderate to severe curvature of the BA and 18 had elongation of the BA. Eighty-six patients had lateral displacement of the BA (⩾ grade 1) and a directional relationship was detected between the dominant VA side and the BA curvature in the opposite direction in 76 (83.5%) patients (76/91; p<0.001).

**Figure 2:**
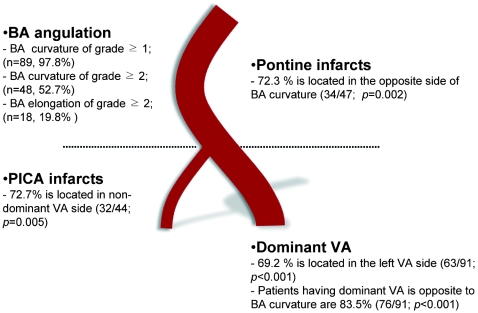
Directional relationships according to BA angulation, VA dominancy and peri-vertebral junctional infarcts (ie, pontine and PICA infarcts). BA, basilar artery, PICA, posterior inferior cerebellar artery; VA, vertebral artery.

Figure 2 shows that pontine infarcts opposite to the side of BA curvature occupied 72.3% (34/47; p = 0.002) while PICA infarcts on the same side of the non-dominant VA occupied 72.7% (32/44; p = 0.005).

### Relationship between diameter difference of VAs and BA dolichosis

Patient demographics according to the diameter difference of the VAs are shown in table 2. The number of patients with hypertension and moderate to severe BA curvature was more and more prevalent towards a higher tertile of VA diameter differences. The mean diameter of the right VA gradually decreased in the higher tertile group. Mean WSSs on the right and particularly on the non-dominant VA increased in the higher tertile group of VA diameter differences.

**Table 2:** Clinical and haemodynamic characteristics in 91 patients stratified by tertiles according to diameter difference of the VAs

	Diameter difference of VAs (mm)			p Value
	Low 1/3 (range 0.04∼0.70)	Middle 1/3 (range 0.71∼1.17)	High 1/3 (range 1.19∼2.67)	
No of cases	30	30	31	
Age (year) (mean (SD))	62.7 (13.0)	62.5 (11.0)	66.2 (10.4)	0.390
Risk factors (n (%))				
Hypertension	17 (56.7)	21 (70.0)	27 (87.1)	0.031*
Diabetes mellitus	11 (36.7)	14 (46.7)	10 (32.3)	0.497
Smoking	10 (33.3)	8 (26.7)	12 (40.0)	0.547
Previous stroke	8 (26.7)	5 (16.7)	5 (16.1)	0.512
Initial laboratory findings (mean (SD))				
Haemoglobin (g/dl)	14.3 (1.6)	13.6 (1.5)	14.4 (1.8)	0.166
Glucose (mg/dl)	137.7 (48.0)	171.0 (74.1)	151.7 (67.1)	0.138
Total cholesterol (mg/dl)	176.0 (36.4)	171.8 (45.8)	180.4 (41.7)	0.984
C reactive protein (mg/dl)	0.36 (0.51)	0.80 (2.18)	0.69 (1.49)	0.512
Fibrinogen (mg/dl)	314.3 (72.6)	357.4 (88.3)	324.9 (74.2)	0.112
Radiological findings				
M-to-S BA curvature (n (%))	10 (33.3)	15 (50.0)	23 (74.2)	0.006*
M-to-S BA elongation (n (%))	4 (13.3)	9 (30.0)	5 (16.1)	0.221
Diameter of BA (mm) (mean (SD))	3.15 (0.58)	3.16 (0.56)	3.27 (0.55)	0.660
Diameter of R VA (mm) (mean (SD))	2.29 (0.38)	1.98 (0.56)	1.58 (0.86)	0.001*
Diameter of L VA (mm) (mean (SD))	2.35 (0.60)	2.52 (0.57)	2.53 (0.97)	0.589
WSS (dyn/cm^2^) (mean (SD))				
WSS in R VA	7.1 (2.0)	11.2 (7.7)	14.0 (8.2)	0.004*
WSS in L VA	6.9 (2.8)	9.1 (8.2)	8.5 (4.4)	0.407
WSS in BA at 80 mm	6.6 (4.0)	8.3 (5.5)	8.2 (4.0)	0.429
WSS in BA at 90 mm	6.4 (5.1)	8.5 (4.8)	8.4 (3.8)	0.308
WSS in dominant VA	6.6 (2.4)	8.1 (5.3)	6.9 (2.6)	0.449
WSS in non-dominant VA	7.4 (2.5)	12.3 (9.5)	15.6 (7.5)	0.001*

BA, basilar artery; L, left; M-to-S, moderate to severe; R, right; VA, vertebral artery; WSS, wall shear stress.

*p<0.05.

Hypertension, older age and the diameter difference of the VAs were associated with moderate to severe BA curvature in univariate analysis. After adjusting for all potential variables, only the diameter difference of the VAs remained as an independent predictor of moderate to severe BA curvature in the full multivariate model (OR per 1 mm increase 2.70, 95% CI 1.22 to 5.98; p = 0.015). Other haemodynamic variables, such as viscosity, other vessel diameters, WSR and WSS of vessels, were not significantly associated with moderate to severe BA curvature.

## Discussion

We found that pontine infarcts more frequently occurred opposite to the side of BA curvature and PICA infarcts were more frequent on the non-dominant VA side, and that the difference in the diameters of the right and left VA was the only independent predictor for moderate to severe BA curvature. Hence the present study suggested that the asymmetrical flow pattern of VAs around the vertebrobilar junction might be an important mechanical force in the origin of BA curvature as well as a causative factor of peri-vertebrobasilar junctional infarcts (ie, pontine and PICA infarcts).

In this study, VA diameter was significantly larger on the left than on the right side, which is consistent with numerous previous reports.^7^ ^13^ ^14^ Traditionally, most physicians have regarded an asymmetric VA as a congenital variant or a clinically meaningless finding, unless vertebrobasilar insufficiency occurs.^15^ ^16^ However, recent studies have regarded VA hypoplasia as a risk factor for posterior circulation stroke, and that its directional association with a stroke had an ipsilateral tendency to VA hypoplasia.^13^ ^17^ ^18^ ^19^ Interestingly, we also found that a specific stroke (ie, pontine infarction) had a tendency to the opposite side to the lateral displacement of the BA. In addition, the curvature of the BA was directionally opposite to the dominant VA.

Vertebrobasilar dolichoectasia is anatomically well known and appears to be postulated as a risk factor for posterior circulation stroke.^20^ ^21^ ^22^ Currently, a study reported on the directional relationship between vertebrobasilar dolichoectasia and the specific locations of infarcts in the vertebrobasilar system—that is, 58% of patients (11/19) had pontine or cerebellar lesions (superior and anterior cerebellar lesions) contralateral to the lateral displacement of the BA versus ipsilateral in 26% of patients (5/19).^20^ A recent study on consecutive series of cerebellar infarcts showed that cerebellar infarcts at the post-vertebrobasilar junction (anterior inferior cerebellar artery and superior cerebellar artery territory) were more common on the left side. This study also indicated that there was a right predilection at the pre-vertebrobasilar junction (PICA territory) and a left predilection at the post-vertebrobasilar junction (superior cerebellar artery territory) even in patients with combined involvements of cerebellar arterial territories.^23^ For these reasons, our findings may provide more evidence that the specific location of infarcts is closely connected to BA curvature or VA hypoplasia.

**Figure 3:**
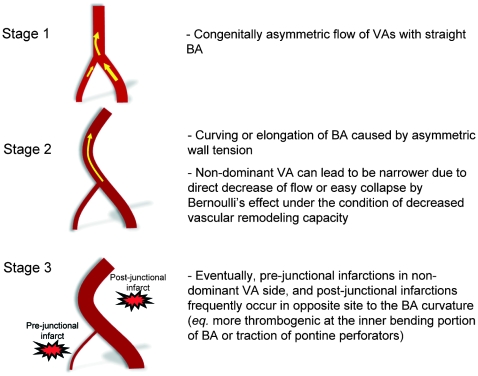
Schematic illustrations of the pathophysiological process of peri-vertebral junctional infarcts: possible changes in vetebrobasilar vessels under the condition of an unequal VA flow. BA, basilar artery, VA, vertebral artery.

Such directional relationships can be explained by several haemodynamic mechanisms. Firstly, the inner wall of the BA curvature may be more thrombogenic because of a low WSS,^4^ ^24^ and traction of the pontine perforators caused by BA curvature may lead to infarction.^20^ Secondly, a hypoplastic VA can cause ipsilateral PICA infarction by directly decreasing blood flow in the smaller intracranial VA.^24^ This can occur because of the easy collapsibility of a narrowed vessel as a result of Bernoulli’s effect under the decreased VA remodelling capacity.^25^ Our theory is outlined in fig 3. The vector of BA flow merging from unequal VAs makes the BA flow curve to the side of the weaker VA, and the chronic processes caused by asymmetric VA flow can induce greater curving of the BA wall. Subsequently, such deformation of the BA can cause atherogenesis, leading to ischaemic stroke in the vertebrobasilar system. A hypoplastic VA can also result in the ipsilateral occlusion of this vessel due to a direct decrease in blood flow and easy collapse of the vessel caused by the smaller intracranial VA calibre.

The diameter difference of the VAs was the only independent predictor for the moderate to severe BA dolichosis, even after adjusting for confounding variables (demographics, radiological variables and haemodynamic variables). In general, the flow rate is proportional to the fourth power of the vessel radius, according to Poiseuille’s law.^24^ Therefore, the radius of the vessel is the most essential determinant of blood flow, even in the vertebrobasilar system. Pressure, blood flow or both factors are widely recognised as potential stimuli for morphological change or the functional adaptation of vessels.^26^ Numerous studies have examined flow dependent remodelling,^26^ ^27^ ^28^ ^29^ and if such haemodynamic forces are altered chronically, a subsequent morphological or structural adaptation of the artery can occur to minimise the effect of the altered haemodynamic forces on the vascular wall, including changes in calibre and wall thickness.

Our study has several limitations. We investigated the values of WSSs using pulsed Doppler sonography. Although it is a simple, easy and non-invasive way for the assessment of WSS, it can be technically limited as it is unable to visualise the vessel and also has an angle correction problem. Additionally, it is necessary to assume a linear velocity distribution of the flow with the central peak velocity to measure the wall shear rate. In future studies, accurate and timely measurement of WSS with good spatial resolution should be used. Further research involving vessel remodelling mediators, such as matrix metalloproteinases and nitric oxide, suggesting a role for vascular adaptation, is needed to assess the regulatory aspects of the vessel in response to the flow related mechanical forces.^27^

In conclusion, this study suggests that unequal blood flow from bilateral VAs is a significant haemodynamic contributor to BA curvature. Moreover, it is a potential determinant for the development of acute infarcts around the vertebrobasilar junction.
